# The Impact of AAPH-Induced Oxidation on the Functional and Structural Properties, and Proteomics of Arachin

**DOI:** 10.3390/molecules28176277

**Published:** 2023-08-28

**Authors:** Mingjuan Shen, Xi Yang, Zhenxing Wang, Xiaomei Sha, Xuechun Zhang, Jian Sun

**Affiliations:** 1 Key Laboratory for Forest Resources Conservation and Utilization in the Southwest Mountains of China, Ministry of Education, Key Laboratory of Forest Disaster Warning and Control of Yunnan Province, College of Life Science, Southwest Forestry University, Kunming 650224, China; shenmingjuan@swfu.edu.cn (M.S.); xi_yang2076@163.com (X.Y.); wangzhenxingfood@163.com (Z.W.); 2 National R&D Center for Freshwater Fish Processing, College of Life Sciences, Jiangxi Normal University, Nanchang 330022, China; 3 Guangxi Academy of Agricultural Sciences, Nanning 530007, China; jiansun@gxaas.net

**Keywords:** arachin, AAPH, oxidation, functional properties, structural characteristics, proteomics

## Abstract

The aim of this study was to investigate the effect of 2,2′-azobis (2-amidinopropane) dihydrochloride (AAPH)-induced oxidation on the functional, structural properties and proteomic information of arachin. The results showed that moderate oxidation improved the water/oil holding capacity of proteins and increased the emulsifying stability, while excessive oxidation increased the carbonyl content, reduced the thiol content, altered the structure and thermal stability, and reduced most of the physicochemical properties. Through LC-QE-MS analysis, it was observed that oxidation leads to various modifications in arachin, including carbamylation, oxidation, and reduction, among others. In addition, 15 differentially expressed proteins were identified. Through gene ontology (GO) analysis, these proteins primarily affected the cellular and metabolic processes in the biological process category. Further Kyoto encyclopedia of genes and genomes (KEGG) analysis revealed that the “proteasome; protein processing in the endoplasmic reticulum (PPER)” pathway was the most significantly enriched signaling pathway during the oxidation process of arachin. In conclusion, this study demonstrated that AAPH-induced oxidation can alter the conformation and proteome of arachin, thereby affecting its corresponding functional properties. The findings of this study can potentially serve as a theoretical basis and foundational reference for the management of peanut processing and storage.

## 1. Introduction

Proteins and lipids are essential nutrients in human nutrition and key components of food systems. During food processing and storage, lipids, particularly unsaturated fatty acids, are prone to oxidation leading to the formation of lipid peroxidation products, including reactive oxygen species (ROS), aldehydes, and free radicals, which in turn induce protein oxidation [1].

Studies have shown that protein oxidation can result in carbonylation, intermolecular disulfide bond formation and aggregation, decreased solubility, and changes in functional properties (e.g., surface hydrophobicity, emulsification, water-holding capacity, texture, flavor, and nutritional value), leading to loss of food nutrition, deterioration of flavor, and decreased functional properties, thereby affecting its quality and safety [2,3,4,5,6,7]. Therefore, investigating protein oxidation is of great significance, and it has become one of the hottest research areas in the field of food.

Owing to the intricate nature of the food system, the free radical oxidation model has emerged as a practical and viable approach to probing protein oxidation [8,9]. Of which, 2,2′-azobis (2-amidopropane) dihydrochloride (AAPH) is a commonly used oxidation model. It has the ability to undergo spontaneous decomposition in an aqueous solution, resulting in the formation of peroxyl radicals (ROO^•^) [10,11]. ROO^•^ can abstract hydrogen atoms from protein, leading to protein chain oxidation reactions [12]. Studies have reported that the ROO^•^-induced oxidation significantly affects the conformation and function of meat and soy proteins, including taste and texture [13,14]. In general, current research primarily focus on ROO^•^-induced protein oxidation in meat, with limited investigation of plant proteins.

Peanut (*Arachis hypogaea* L.) is recognized as a high-quality food ingredient due to its protein content of up to 22–30%, lack of anti-nutritional factors, and complete amino acid profile, as well as its distinctive flavor [15]. Arachin, which accounts for about 44.3–62.7% of the total content of peanut protein, plays a crucial role in the thermal, immune, allergenic, and physicochemical properties of peanut protein while functioning as an essential storage protein during seed development and germination [16,17,18]. The high unsaturated fatty acid content in peanut kernels makes peanut protein highly susceptible to oxidation. Given the immense value of peanuts in the food industry, investigating the oxidation of peanut protein, particularly arachin, is imperative. Despite there being some studies on the oxidation of peanut proteins, research on the effects of oxidation on the functional properties of arachin and their underlying mechanisms are still very limited.

Therefore, this study used AAPH to induce the oxidation of arachin, and evaluated the effects of oxidation on the structural characteristics, processing properties, and oxidative modifications of arachin. Additionally, it speculated on the mechanisms of action of oxidation using mass spectrometry (MS)-based proteomics. This study could provide a theoretical basis for investigating the changes of peanuts during storage and processing, especially for the control and practical utilization of peanut protein oxidation.

## 2. Results and Discussion

### 2.1. Oxidation Degree

**Figure 1:**
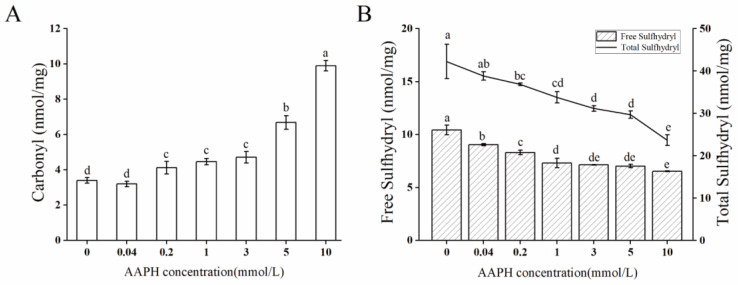
Oxidation index of arachin after oxidation at different AAPH concentrations. (**A**) Carbonyl content; (**B**) sulfhydryl content. AAPH = 2,2′-azobis(2-aminopropane) dihydrochloride. Different letters in the columns indicate significant differences (*p* < 0.05).

Carbonylation is the most common oxidative protein damage, and carbonyl content is a representative indicator [19]. Therefore, the carbonyl and sulfhydryl contents were used to characterize the degree of oxidation in this study. As illustrated in Figure 1A, the concentration of AAPH is directly related to the increase in the carbonyl content of arachin, and the highest carbonyl content of 9.894 nmol/mg was observed with an AAPH concentration of 10 mmol/L. Our previous investigation showed a comparable outcome [20]; this is because reactive oxygen species (ROS^•^) and protein molecules can generate radicals on the side chains of amino acids or the main chains through hydrogen abstraction reactions under aerobic conditions. These radicals react to form carbonyl derivatives, including aldehydes and ketones [21]. As shown in Figure 1B, the contents of total sulfhydryl and free sulfhydryl decreased significantly with the increase in AAPH concentration (*p* < 0.05). When the AAPH concentration was 10 mmol/L, the total sulfhydryl and free sulfhydryl contents reached the lowest values of 5.534 and 23.665 nmol/mg. The reason is that peroxide radicals (ROO^•^) can rapidly react with the sulfhydryl groups in protein to form mercaptol peroxides or irreversible sulfonic and sulfonic acid groups, ultimately resulting in the reduction of the sulfhydryl groups [20,21]. These results are also consistent with the carbonyl analysis results mentioned above.

### 2.2. Physicochemical Properties

Solubility is a crucial factor in assessing the functional properties of food proteins. It is a broad measure of a protein’s hydrating ability and is commonly used to indicate the extent of aggregation and denaturation of oxidized proteins [1]. Table 1 illustrates that arachin has a relatively high solubility in phosphate-buffered saline [22]. Treatment with AAPH resulted in a significant decrease in the solubility of arachin (*p* < 0.05). At a 10 mmol/L concentration of AAPH, the solubility of arachin reduced to its lowest value of 65%. This is due to the formation of soluble aggregates when the protein is exposed to a low concentration of AAPH. As the concentration of AAPH increases, these soluble aggregates further form insoluble aggregates via covalent cross-linking, leading to a decrease in solubility [23].

As shown in Table 1, the WHC and OHC of arachin exhibited an initial increment followed by a decreasing tendency with AAPH concentration. At an AAPH concentration of 3 mmol/L, both the WHC and OHC reached maxima (maximum values) of 2.98 and 8.25 g/g. These results could be due to the gradual loosening of the structure of arachin and an increase in porosity as the oxidation degree increased, which, in turn, resulted in an increase in the water contact area and water uptake [24]. In addition, our previous study found that the oil absorption capacity was closely related to the hydrophobic groups on the surface of the protein [20]. Therefore, based on the above results, we can conclude that mild oxidation could induce enhanced exposure of hydrophobic groups, thereby increasing the protein’s capacity for absorbing oil. While vigorous oxidation could predispose the protein to aggregate and reduce its oil holding capacity.

The emulsifying activity index (EAI) represents the ability of a protein to form emulsions via absorbing at the oil–water interface, while the emulsifying stability index (ESI) refers to the stability of the emulsion over a specified period of time [25]. From Table 1, we observe that both EAI and ESI exhibited an initial increase followed by a subsequent decrease. The maximum EAI value was found to be 0.35 m^2^/g at an AAPH concentration of 10 mmol/L, while the maximum ESI value was determined to be 20.09 min at a 3 mmol/L AAPH concentration. The reason could be that sulfhydryl groups are oxidized to form disulfide bonds at appropriate concentrations of AAPH, resulting in the cross-linking of the protein that contributes to the formation of more stable protein films on the surface of oil droplets, which ultimately manifests as an increase in both EAI and ESI [26].

### 2.3. Denaturation Temperature

The thermodynamic properties play a crucial role in defining the characteristics of proteins. As proteins absorb or release heat, various changes in the structure and conformational of the protein (primarily the tertiary structure) occur, including the breaking of hydrogen bonds, disruption of hydrophobic interactions, and aggregation, ultimately resulting in modified functional properties [27,28]. Table 1 presents the thermodynamic properties of arachin for different treatment groups. As the AAPH concentration increased, the denaturation enthalpy (∆H) and denaturation temperature (T_d_) of arachin first increased and then decreased. The results showed that arachin underwent aggregation when AAPH concentrations were lower than 1 mmol/L. This resulted in an increase in thermal and tertiary conformational stability [29]. Consequently, the denaturation temperature progressively increased. Once the AAPH concentration exceeded 1 mmol/L, the aggregated structure of arachin was destroyed; as a result, the denaturation temperature decreased.

### 2.4. Structural Characteristics

**Figure 2:**
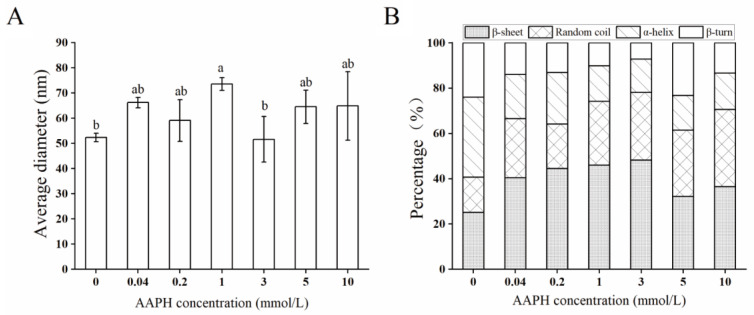
Structural characteristics of arachin oxide at different AAPH concentrations. (**A**) Average diameter; (**B**) secondary structural unit components. AAPH = 2,2′-azo(2-aminopropane) dihydrochloride. Different letters in the columns indicate significant differences (*p* < 0.05).

The particle size is often used to characterize the degree of protein aggregation [30]. As shown in Figure 2A, the average diameter of arachin significantly increased (*p* < 0.05) and reached the maximum value (73.57 μm) at 1 mmol/L AAPH. The reason might be that oxidation made the hydrophobic groups of arachin exposed, which further refolded and assembled through intermolecular forces, and then formed large protein aggregates [20,31]. However, when the concentration reached 3 mmol/L, the average diameter showed a decreasing tendency. It is possible that some of the aggregates break down into smaller soluble peptides [1], which could further convert into insoluble interactions but also lead to a decrease in arachin solubility. This is consistent with the study conducted by Huang et al. [32].

Amide band I in the infrared spectrum is often used for protein secondary structure analysis. For example, the peaks at 1615–1637, 1682–1700, and 1637–1645 cm^−1^ are generally characteristic of β-sheet and random coil, whereas peaks at 1646–1664 and 1646–1681 cm^−1^ are typical of α-helix and β-turn [33] For arachin oxide at different AAPH concentrations, their secondary structural unit components are shown in detail in Figure 2B, and their corresponding Fourier transform infrared spectroscopy (FTIR) spectra and fitting of the secondary structure region are illustrated in Appendix. From Figure 2B, a significant reduction in the percentage of α-helix structure was observed, whereas the percentage of random coils was significantly increased with increasing AAPH concentration (*p* < 0.05). This indicates that the regularity in the arachin peptide chain structure was disrupted due to the attacks of ROO^•^, which in turn induced structural rearrangement through hydrophobic interactions and disulfide bonds [34]. This is also consistent with our previous study, where the protein structures were changed from ordered to disordered to a certain extent by oxidative modification, which is detrimental to the secondary structure maintenance [20].

**Figure 3:**
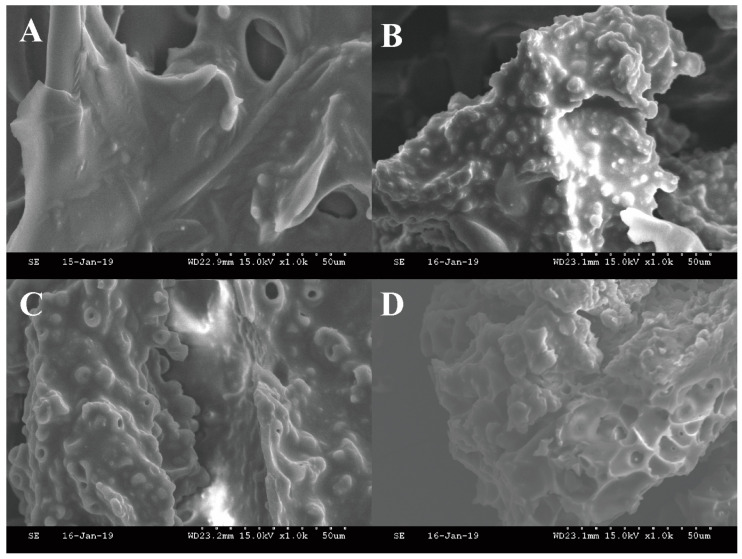
Scanning electron microscope (SEM) of arachin at AAPH concentration of 0, 0.2, 3, and 10 mmol/L (**A**–**D**). All photos are magnified 1000 times. AAPH = 2,2′-azobis (2-amidinopropane) dihydrochloride.

Scanning electron microscopy (SEM) was employed at 1000× magnification to investigate the impact of oxidation on the surface structure of arachin. The unoxidized arachin surface was found to be smooth and flat, as illustrated in Figure 3A. With an increase in AAPH concentration to 10 mmol/L, the arachin surface structure became a rough and uneven surface (Figure 3D). These results provide further confirmation that ROO^•^ leads to the aggregation of arachin into larger particles; a previous study on the oxidative effects of porcine myofibrillar proteins has also reported similar result [30].

### 2.5. Proteomic Analysis

Proteomic analysis was conducted using nano liquid chromatography-Q exactive mass spectrometry (LC-QE). A total of 5198 peptides were detected. Among them, 2289 protein groups were quantified corresponding to 3677 unique peptide sequences (see Supplementary Table S1 for details).

A thorough analysis of amino acid sidechain modifications was conducted based on their respective amino acid sequence. The outcomes are outlined in Table 2, demonstrating the detection of a total of 16 different modifications, including carbamidomethyl, oxidation, dioxidation, dehydroalanine, dehydration, trioxidation, 4-hydroxy-2-nonenal (HNE), kynurenin, hydroxykynurenine, γ-glutamic semialdehyde (GGS), carboxyethylation, carbonylation, acetyl, α-aminoadipic semialdehyde (AAS), α-aminoadipic acid (AAA), and melondialdehyde (MDA).

After analyzing the data, Lysine was identified as the most commonly modified amino acid, with seven types of side-chain modifications. Cysteine and tryptophan were closely behind with five modifications, while histidine, tyrosine, and arginine had four, phenylalanine had three, methionine and proline both had two, and serine had only one. Of these modifications, sulfhydryl groups (-SH) on cysteine residues were found to be particularly vulnerable to oxidative changes. These modifications can result in the oxidation of cysteine sulfonic acid (-SO_3_H) via reactive oxygen species (-ROS), hydrogen peroxide (H_2_O_2_), while forming disulfides (-SS-) and mixed disulfides [35]. Oxidative post-translational modifications (OxiPTMs) of cysteine residues are closely linked to the signal pathways that regulate sulfhydryl redox and consequently affect cell proliferation, differentiation, and migration [36]. Furthermore, we discovered that AAS, GGS, MDA, and other amino acid modifications listed in the table, as well as aldehydes, ketones and other substances, are generated by the oxidation of corresponding amino acids in arachidonic acid, consistent with the product characteristics of protein oxidation reactions [28].

**Figure 4:**
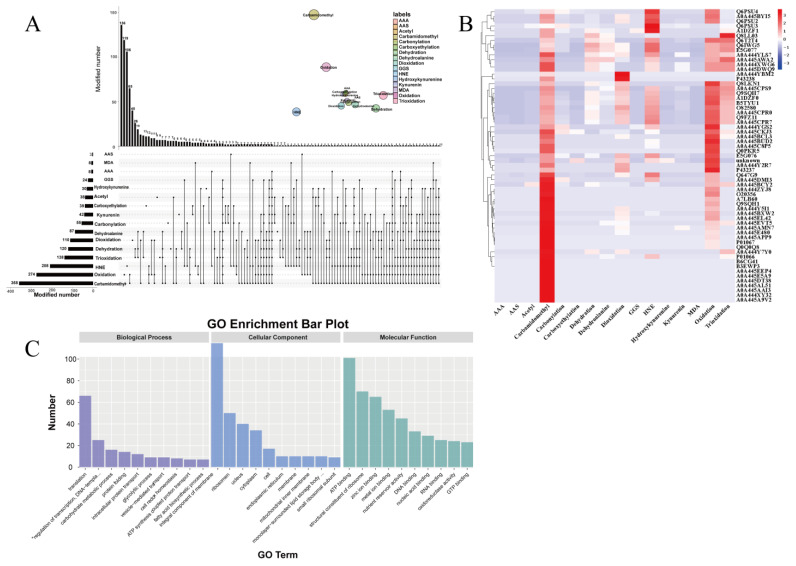
Proteomic analysis of oxidative modifications of arachin. (**A**) UpSet plot of interactions between different modifications and Venn diagram of interactions between different modifications. (**B**) Modification clustered heatmap of different protein groups (modification number ≥ 5). (**C**) The top 10 functional enrichment analyses of gene ontology (GO).

To investigate the distinctions and links among several types of modifications, we constructed advanced Venn diagrams illustrated in Figure 4A. Five specific modifications, namely carbamidomethyl, oxidation, HNE, trioxidation, and dehydration, were discovered to have the highest occurrence frequencies, which were 358, 274, 208, 138, and 120, respectively. Among them, 512 single modifications occurred, while composite modifications occurred 344 times.

To represent the degree of modification of different protein groups, we generated a heat map using oebiotech online website (https://cloud.oebiotech.cn/task/, accessed on 13 August 2022), showing log10 values of the number of modifications (≥5). In descending order, the most common types of modification for these protein groups are oxidation, carbamidomethyl, HNE, trioxidation, and deoxidation, which is consistent with the results shown above in Figure 4B. The top ten protein groups with the highest number of modifications are A0A445CPR0 (320 modifications), Q9FZ11 (308 modifications), A0A445CPR7 (303 modifications), Q6IWG5 (298 modifications), B5TYU1 (290 modifications), A1DZF0 (286 modifications), Q6T2T4 or Q9SQH7 (263 modifications), E5G077 (255 modifications), and Q8LKN1 (248 modifications). According to the UniProt database (http://www.uniprot.org, accessed on 23 August 2022), the aforementioned proteins are the sources of sulfur-containing amino acids, which can be found in plant seeds, with the exception of Q9SQH7 (Glycine), E5G077 (Ara h 3 allergens), and Q8LKN1 (Ara h 3 or Ara h 4 allergens) as indicated by Wang et al. [37], during oxidation, the active-site cysteine in arachin was oxidized to a sulfenic acid intermediate and subsequently formed an intermolecular disulfide bond with another active site cysteine. This finding explains why sulfur-containing amino acids had the most frequent modifications. Moreover, AAPH-induced oxidation up-regulated mono-oxidation and tri-oxidation modifications of E5G077 and Q8LKN1, which belong to the peanut allergen gene. This speculates that oxidation might reduce the allergenicity of peanuts through the modification of related protein groups.

We further identified 2289 proteins through mass spectrometry analysis of the aforementioned peptides, and the details are shown in Supplementary Table S2. In order to gain insight into the functions of these proteins, we performed Gene Ontology (GO) annotations (http://geneontology.org/page/go-annotation-conventions, accessed on 13 April 2022). Through analysis, 961 GO terms have been annotated, including 173 terms in biological processes, 468 in molecular function, and 320 terms in cellular components. For each classification, we displayed the top 10 enriched GO terms in Figure 4C. Of them, the most enriched biological process was “translation” (66), whereas the most significantly enriched molecular function was “ATP binding” (101), and the most enriched cellular component was “integral components of membranes” (130). In general, “translation” primarily encompasses the adenylation of amino acids, tRNA charging, and peptide bond formation, while post-translational modifications (PTMs) may be viewed as chemical switches capable of influencing protein interactions [35]. As such, unraveling the primary biological functions of these proteins remains of great interest.

**Figure 5:**
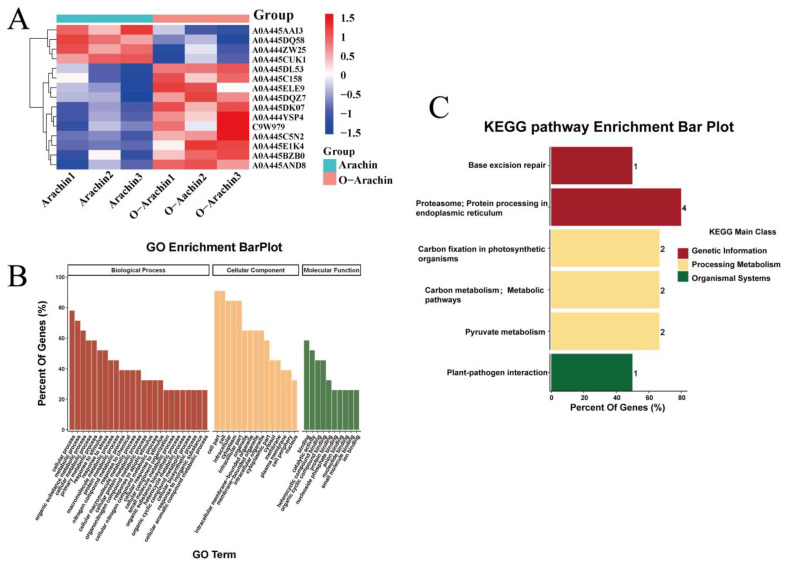
Analysis chart of differentially expressed proteins (DEPs) of arachin. (**A**) The differentially expressed proteins (DEPs) between arachin and oxidized arachin (O-arachin). (**B**) GO enrichment analysis based on DEPs. (**C**) Enrichment map of signaling pathways via the KEGG pathway database.

We determined the proteins that were affected by oxidation through the identification of differentially expressed proteins (DEPs) between arachin and oxidized arachin (O-arachin) via quantitative mass spectrometry-based protein profiling, and the results are provided in Supplementary Table S3. A total of 15 proteins were significantly differentially expressed, based on the screening criteria (*p* < 0.05, FC ≥ 1.5 or <−1.5), as illustrated in Figure 5A. Among them, four proteins showed significant down-regulation with oxidation treatment, while 11 showed significant up-regulation. Overall, the significantly altered proteins are primarily involved in energy metabolism, protein structure, and some uncharacterized proteins. These changes may be responsible for the alteration of the function and structure of arachin.

Further gene ontology (GO) analysis was conducted based on the DEPs and the results were presented in Figure 5B, which revealed the most common categories of biological processes to be “cellular process”, “metabolic process”, and “organic substance metabolic process”. Meanwhile, the most common categories of cellular components included “cell part”, “cell”, and “intracellular”. As for molecular function, the categories that exhibited the highest levels of enrichment were “binding”, “catalytic activity”, and “heterocyclic compound binding”. Our research also found that up-regulated proteins were mainly enriched in biological processes, whereas down-regulated proteins mainly belonged to the molecular function.

To further investigate the signaling pathways for the DEPs, we conducted Kyoto encyclopedia of genes and genomes (KEGG) enrichment analysis utilizing the KEGG pathway database (http://www.genome.jp/kegg/, accessed on 21 August 2022). From Figure 5C, we found that a total of six signaling pathways were enriched. The pathway “proteasome; protein processing in the endoplasmic reticulum”, which belongs to the “folding, sorting and degradation” subclass, had the highest enrichment level (4 genes enriched), followed by “pyruvate metabolism”, “carbon metabolism; metabolic pathways”, and “carbon fixation in photosynthetic organisms” pathways, enriched a total of 2 genes each, belonging to the pathway subclasses of “carbohydrate metabolism”, “energy metabolism”, and “global and overview maps”, respectively. “Base excision repair” and “plant-pathogen interaction” each enriched 1 gene and belonged to the subclass “replication and repair” and “environmental adaptation”, respectively. The aforementioned KEGG results, particularly the high level of the gene enrichment in the “proteasome, protein processing in endoplasmic reticulum” pathway (see Supplementary Figure S1), provide further insight into the underlying mechanisms of protein structural and functional changes following the oxidation treatment.

## 3. Discussion

Peanuts, which served as a valuable food source abound with proteins and unsaturated fatty acids, are susceptible to lipid oxidation during processing and storage, which in turn leads to protein oxidation in peanuts. Arachin plays a significant role in peanut protein; however, there is a lack of research on how protein oxidation affects their function and structure. In this study, an oxidative model for peanut globulin was established by using AAPH, which produced peroxyl radicals, and the impact of the AAPH-induced oxidation on arachin was analyzed.

The results demonstrate that oxidation has a significant impact on both the structure and function of arachin. This impact is specifically observed as follows: As the degree of oxidation deepens, the carbonyl content of arachin gradually increases (from 3.629 to 9.894 nmol/mg), while the levels of both total sulfhydryl and free sulfhydryl show a corresponding gradual decrease (23.665 and 5.534 nmol/mg, respectively). The reason may be that the high activity of free radicals enables proteins to be oxidized directly, which causes the breakage of polypeptide chains and the formation of carbon based cross-links via covalent bonding with side-chain free radicals [17]. Therefore, these indicators can serve as important markers in reflecting the degree of protein oxidation [19,38,39].

Furthermore, excessive oxidation can lead to a decrease in the denaturation temperature of arachin, aggregation, an increase in particle size, a decrease in solubility, and disruption of the secondary structure. Interestingly, it was observed that the proteins demonstrate an improvement in their ability to hold oil and water in the early stages of the oxidation process. This is because mild oxidation increases the exposure of hydrophobic regions, resulting in the folding and assembly of these regions, forming large protein aggregates [20,24]. In contrast, intense oxidation disrupts the regularity of the protein’s secondary structure, resulting in the unfolding and rearrangement of peptide chains, making the protein more prone to aggregating, and reducing the protein’s ability to hold oil and water [30]. 

In addition to the physicochemical properties and structure of arachin, we also evaluated the impact of AAPH-induced oxidation on its proteome. By using proteomics, the amino acid side-chain modifications were examined based on their respective amino acid sequences, and 16 distinct modifications were detected, with lysine being the most commonly modified amino acid. One additional interesting result was that the mono-oxidation and tri-oxidation modifications of E5G077 and Q8LKN1, two peanut allergen genes, were up-regulated. Previous studies have demonstrated that oxidation has a comparable impact on the antigenicity of walnut proteins [20]. Moreover, with the increase in AAPH concentration, the antigenicity gradually decreases. The reason may be that oxidative modifications might induce alterations in the protein side chains, resulting in the disruption of walnut protein’s allergenic epitopes, ultimately reducing their allergenicity. Corresponding outcomes were observed in both acrolein-oxidized shrimp tropomyosin [40] and hydroxyl radical-treated β-conglycinin [41]. This reveals the potential for reducing protein sensitization through oxidative modification. 

Through the GO and KEGG enrichment analysis of differentially expressed proteins before and after oxidation, it was found that the main protein functions affected by oxidation are energy metabolism and protein structure, primarily focusing on signal pathways related to protein processing. These may be the possible mechanisms underlying the changes in the function and structure of arachin after oxidation.

## 4. Materials and Methods

### 4.1. Materials

Peanut kernels were purchased from the local market in Kunming, China. The purchased 2,2′-azobis (2-amidinopropane) dihydrochloride (AAPH), 2,4-dinitrophenylhydrazine (DNPH), 2-nitrobenzoic acid (DTNB), disodium ethylenediamine tetraacetate (EDTA), sodium dodecyl sulfate (SDS) and other reagents were of analytical purity.

### 4.2. Sample Preparation

#### 4.2.1. Extraction of Arachin

Arachin was extracted from peanut kernels using the alkali dissolution-acid precipitation method, following Zhao et al. [42]. Degreasing peanut kernels were dispersed in Tris-HCl buffer (pH 7.9, 1:10 *w*/*v*), and then stirred for one hour. Next, the mixture was centrifuged at 4000× *g* and at 4 °C for 10 min. The resulting precipitation was centrifuged under the same conditions, and washed with deionized water until neutral, then freeze-dried for future use. The extract was identified as arachin using a polyacrylamide gel system analysis.

#### 4.2.2. Oxidation of Arachin

The modified method of Li et al. [17] was used for the oxidation experiment. A protein solution with a concentration of 10 mg/mL was prepared using a 0.01 mol/L pH 7.4 phosphate buffer. Different concentrations of AAPH (0, 0.04, 0.2, 1, 3, 5, and 10 mmol/L) were added to the protein solution. After incubation at 37 °C in the dark for 24 h, the mixture was cooled to 4 °C and finally dialyzed at 4 °C for 72 h. Next, the dialysates were freeze-dried at −80 °C for 24 h to obtain AAPH-oxidized arachin and were stored at −80 °C for future use.

### 4.3. Determination of Carbonyl Content

The carbonyl content was analyzed according to the method of Patrícia et al. [38]. Incubate 0.45 mL protein dispersion (10 mg/mL) with 0.3 mL DNPH solution (10 mmol/L, containing 2 mol/L HCl) in the dark at room temperature for 1 h. Next, add 0.3 mL of Trichloroacetic acid (TCA, 20%) and mix well. Centrifuge the mixture at 10,000× *g* for 10 min. Wash the precipitate thrice with 0.3 mL ethyl acetate–ethanol solution (1:1, *v*/*v*). Then, dissolve it in 0.6 mL urea (6 mol/L) for 15 min at 37 °C. After centrifuging the mixture at 10,000× *g* for 10 min, measure the absorbance of the supernatant at 370 nm with a microplate reader (BIOTEK, MD SpectraMax Plus 384, Silicon Valley, CA, USA). Express the protein carbonyl content as nmol/mg using a molar extinction coefficient of 22,000 L/(mol·cm) ^−1^.

### 4.4. Determination of Sulfhydryl Group Content 

The working solutions were prepared for the determination of sulfhydryl groups using the following procedures. First, solution A was prepared by mixing 1.0418 g Tris, 0.6756 g glycerin, and 0.148 g EDTA with 100 mL of pH 8.0 Tris-Gly buffer. This solution was used to determine the amount of free sulfhydryl groups. Second, solution B was created by adding 4.8048 g urea to 50 mL of Tris-Gly buffer and used to determine the total sulfhydryl groups. Finally, solution C was prepared by adding 4 mg/mL of DTNB to the solution A. Both solutions A and B, containing 3 mg/mL of protein each, were prepared, respectively. Then, 1 mL of solutions A and B were mixed with 10 µL of solution C and kept for 1 h at room temperature. After centrifugation at 10,000× *g* for 10 min, the absorbance of the supernatant was determined at 412 nm. The reference was a sample without DTNB and without protein. The sulfhydryl groups in protein samples were then calculated using an extinction coefficient of 13,600 L/(mol·cm) ^−1^ [39,43].

### 4.5. Solubility

The protein was dissolved in a phosphate-buffered solution (0.01 M, pH 7.4) and adjusted to a final concentration of 1 mg/mL. Subsequently, it was centrifuged at 8000× *g* for 5 min at 4 °C. The protein concentration in the supernatant was measured using the Coomassie Brilliant Blue technique, each replicate was determined three times, and the solubility was calculated as the percentage of the soluble protein (mg) compared to the total protein sample (mg) [1].

### 4.6. Water/Oil Holding Capacity (WHC/OHC)

A total of 200 mg of protein powder was mixed with either 10 mL of water or soybean oil, using a vortex (Changzhou Yuexin Instrument Manufacturing Co., Ltd., Chang zhou, China) with a weighted centrifuge tube for 1 min. After centrifugation at a speed of 3000× *g* for 10 min, the supernatant was discarded, and the precipitate was weighed. WHC/OHC was expressed as the amount of water or oil absorbed per gram of protein sample [44].

### 4.7. Emulsifying Activity Index (EAI) and Emulsifying Stability Index (ESI)

The protein samples were suspended in a phosphate buffer (0.05 mol/L, pH 7.0) to achieve a final concentration of 1 mg/mL. Then, 8 mL of the suspension was mixed with 2 mL soybean oil and homogenized at a speed of 10,000× *g* for 1 min. After homogenization, 50 μL of the emulsion was pipetted and thoroughly mixed with 5 mL of SDS solution (0.1%, *w*/*w*). The absorbance was immediately measured, and then again after 10 min at 500 nm, using the SDS solution as the blank. The EAI and ESI of the protein samples were calculated according to their respective equations [33,45].

(1) $$EAI(m^{2}/g)=\frac{2\times 2.303\times A_{0}\times N}{C\times \varphi \times L\times 100,000}$$

(2) $$ESI(min)=\frac{A_{0}}{A_{0}-A_{10}}$$

where *A*_0_ was the absorbance at the moment of 0 min, *N* was the dilution factor (500), *C* was the protein concentration (g/mL), *φ* was the oil volume fraction (25%) used to prepare the emulsion sample, *L* was the Colorimetric Path (1 cm), and *A*_10_ was the absorbance at the moment 10 min.

### 4.8. Particle Size Distributions 

First, the protein sample was suspended in distilled water to obtain a solution with a concentration of 5 mg/mL. The size distribution of particles and average diameter of the sample solution were measured using the LA-960 Particle Size Analyzer from Partica (Kyoto, Japan). The refractive index of the solution was measured at 1.141 and its absorbance was 0.001 [30].

### 4.9. Fourier Transformed Infrared Spectroscopy (FT-IR)

The protein sample powder was freeze-dried, ground and mixed with potassium bromide at a ratio of 1:100 (*w*/*w*). The mixture was pressurized at 22 MPa for 10 s. The proportions of each secondary structure (α-helix, β-sheet, β-turn, and random coil) were analyzed using an FT-IR spectrometer (Thermo Fisher Scientific, Nicolet IS50, Waltham, MA, USA) with a resolution of 4 cm^−1^, and 32 scans taken between 400–4000 cm^−1^. Peakfit Version 4.12 software was utilized to perform Gauss split-peak fitting on the second-order derivative spectra. Baseline correction was applied at 1600–1700 cm^−1^. Then, the ratio of the subpeak area to the total peak area was calculated for each region, based on the secondary structure partitioning [46].

### 4.10. Scanning Electron Microscope (SEM)

The protein powder samples were glued onto the Scanning Electron Microscope (SEM) sample stage, then were wetted and dispersed evenly in absolute ethanol. After applying gold-spray treatment, the SEM images were recorded at different magnifications with the high-resolution scanning electron microscope (S-3000N SEM, Hitachi, Japan) [47].

### 4.11. Determination of Differential Scanning Calorimetry (DSC)

The thermodynamic properties of the samples were characterized using a Differential Scanning Calorimetry (DSC) instrument (204F1 differential calorimetry scanner, NETZSCH-Gerätebau GmbH, Selb, Germany). An empty aluminum box was used as a blank control. The scanning range was set at 25–125 °C with a heating rate of 10 °C/min. The thermogravimetric (TG) curves were plotted using the heat absorption or release rate (unit of sample mJ/s) as the ordinate and the temperature as the abscissa [48].

### 4.12. LC-MS/MS

The Mass Spectrometry analysis was conducted using the Nano-Liquid Chromatography-Q-Exactive mass spectrometer (Thermo Fisher Scientific, Q Exactive, Waltham, MA, USA). Initially, the samples were successively reduced and alkylated. Trypsin was added at a ratio of 1:50 (*w*/*w*) and incubated for 20 h at 37 °C. The enzymatic hydrolysate was desalted, lyophilized, and redissolved in a sequence of formic acid solution (FA, 0.1%, *w*/*w*), and stored at −20 °C for further analysis via Liquid Chromatography (LC). Solution A was an aqueous solution of 0.1% formic acid; and Solution B was composed of 84% acetonitrile and 0.1% formic acid. After achieving equilibrium, the hydrolysate was loaded onto the trap column using an automatic sampler machine. A full scan was performed. Then, 20 fragment profiles were collected to determine the mass/charge ratios of peptides and peptide fragments. The mass spectrometry data obtained were searched against the UniProtKB database using the Proteome Discoverer software (version 1.4; Thermo Fisher Scientific, Waltham, MA, USA) [49].

The raw file was searched against the protein database uniport_Juglans_regia_52259_20211129.fasta using the MaxQuant software (version 1.6.2.0). The protein sequence database consists of 52,259 sequences (downloaded on 29 November 2021 from http://www.uniprot.org, accessed on on 23 August 2022). The search parameters were set as follows: enzyme was set as Trypsin; missed cleavage sites were set to 2; the fixed modification was carbamidomethyl, oxidation, dioxidation, dehydroalanine, dehydration, trioxidation, 4-hydroxy-2-nonenal (HNE), kynurenin, hydroxykynurenine, γ-glutamic semialdehyde (GGS), carboxyethylation, carbonylation, acetyl, α-aminoadipic semialdehyde (AAS), α-aminoadipic acid (AAA), and melondialdehyde (MDA). Proteins identified through database searching had to pass the set filtering parameter of False Discovery Rate (FDR) ≤ 0.01.

### 4.13. Statistical Analysis

All the above experiments were performed in triplicate and repeated three times. Significance analysis was conducted with a one-way analysis of variance (ANOVA). The significance level was set to *p* < 0.05 in IBM SPSS Statistics 25 software (version 25). Graphs were produced by Origin software (version 2021).

## 5. Conclusions

This study investigated the impact of AAPH-induced oxidation on arachin, focusing on its functional, structural, and proteomic aspects. The results indicated that the oxidation induced changes in the main functional groups, structural characteristics, as well as the functional properties of arachin. It is important to note that appropriate oxidation enhances the emulsifying properties as well as the water-holding capacity and oil-holding capacity (WHC/OHC) of arachin. Nevertheless, excessive oxidation can greatly reduce its functional properties. During the oxidation process, many modifications of amino acid side chains have been discovered. These modifications are closely associated with the changes observed in the functional and structural characteristics of arachin investigated in this study. The KEGG analysis identified the “proteasome; protein processing in endoplasmic reticulum” signaling pathway as the most significantly enriched pathway among the six identified pathways. Considering its role in protein folding, sorting, and degradation, this pathway is likely the primary cause for the significant changes in protein structure resulting from oxidation. The findings of this preliminary study offer valuable insights into protein oxidation and provide a basis for the processing and storage of peanut products.

## Figures and Tables

**Table 1:** Effects of different concentrations of AAPH on the properties of arachin.

	Properties	AAPH (mmol/L)						
		0	0.04	0.2	1	3	5	10
Functional	Solubility (%)	94 ± 4 a	89 ± 7 ab	84 ± 7 b	71 ± 2 c	0.68 ± 0.05 c	0.66 ± 0.02 c	0.65 ± 0.01 c
	WHC (g/g)	1.73 ± 0.08 d	1.67 ± 0.11 d	1.79 ± 0.07 d	2.39 ± 0.02 c	2.98 ± 0.16 a	2.47 ± 0.19 bc	2.81 ± 0.31 ab
	OHC (g/g)	7.81 ± 0.21 a	8.09 ± 0.99 a	7.98 ± 1.03 a	7.24 ± 0.36 a	8.25 ± 0.53 a	6.82 ± 0.15 ab	5.50 ± 0.48 b
	EAI (m^2^/g)	0.13 ± 0.01 d	0.14 ± 0.01 d	0.18 ± 0.00 cd	0.26 ± 0.02 abc	0.25 ± 0.09 bc	0.28 ± 0.02 ab	0.35 ± 0.00 a
	ESI (min)	12.411 ± 0.533 c	12.631 ± 0.800 c	12.994 ± 1.004 bc	15.823 ± 0.133 abc	20.093 ± 3.989 a	17.365 ± 2.683 ab	15.077 ± 0.601 bc
Thermographic	Onset denaturation temperature (T_o_/°C)	39.05 ± 0.212	39.45 ± 1.485	41.40 ± 0.707	43.53 ± 1.127	37.97 ± 0.321	38.15 ± 1.909	35.25 ± 0.778
	Denaturation temperature (T_d_/°C)	64.37 ± 3.000	76.70 ± 4.952	76.60 ± 1.300	73.70 ± 7.862	60.9 ± 3.818	62.83 ± 4.761	68.90 ± 3.111
	Maximum denaturation temperature (T_m_/°C)	102.80 ± 2.179	114.07 ± 3.166	105.65 ± 4.596	109.13 ± 5.498	100.25 ± 1.061	101.85 ± 5.445	105.10 ± 1.838
	Denaturation enthalpy (∆H∙J^−1^∙g^−1^)	101.21 ± 11.158	105.57 ± 8.393	91.17 ± 5.616	102.16 ± 23.967	81.14 ± 1.308	94.43 ± 11.413	95.62 ± 3.500

AAPH = 2,2′-azobis (2-amidinopropane) dihydrochloride, WHC = Water Holding Capacity, OHC = Oil Holding Capacity, EAI = Emulsifying Activity Index, ESI = Emulsifying Stability Index, Different letters within a column indicate significant differences (*p* < 0.05).

**Table 2:** Amino acid modification sites and types.

Amino Acid	Sequence			Modification type													
		Carbamidomethyl	Oxidation	Deoxidation	Dehydroalanine	Dehydration	Trioxidation	HNE	Kynurenin	Hydroxykynurenine	GGS	Carboxyethylation	Carbonylation	Acetyl	AAS	AAA	MDA
Cysteine	NAVMAPHYNLNCHAVIYGTEGR	√															
Cysteine	ANLRPCEEHIRQRVEQEQEQEQDEYPYIQR		√														
Cysteine	NSDCQPCCEGFFCPPGLTCMIPCPLGAYCPR			√													
Cysteine	EEGALLCLHCCHLFAFCCRR						√										
Cysteine	QCPNELRVSNR							√									
Methionine	GAPIIAEYLGGAVNCDAYHMTDPR		√														
Methionine	EDECDICLEPCTKMVLPNCCHAMCIR			√													
Proline	CCVSFSAFFNESVVPCQTCACGCSAKPER		√														
Proline	IVPIAER			√													
Phenylalanine	CCVSFSAFFNESVVPCQTCACGCSAKPER		√														
Phenylalanine	CMCEALQQIMENQCDRLQDRQMVQQFK			√													
Phenylalanine	EEGALLCLHCCHLFAFCCRR						√										
Histidine	CNAPCLRCNGHDQRCMAPCLR							√									
Histidine	MCAFCISLCSAEFCSTPPCSSAPLCSSPR			√													
Histidine	CFFLSFVGCHLFYFCCHAIICPSGLAAFESCPITR		√														
Histidine	CQGMTLENVSINFSGSFGVGMVYTALSR						√										
Lysine	CKCRSIGGSCGPSCGCK																√
Lysine	AYEELHQEDLIK							√									
Lysine	CVKELFHFEEDSGGIIK													√			
Lysine	CCAKCLCVPPGYYGNK												√				
Lysine	ALCKSNETIDVALQIFREMPNR															√	
Lysine	CACCGATSTKSSVSK														√		
Lysine	KGSEEGDITNPINLR											√					
Tyrosine	DDVELVAVNDPFITTDYMTYMFK		√														
Tyrosine	NENQNLVLAYLFIPLTTPGDYK				√												
Tyrosine	YSLKPLVPRLTELLGVDVK			√													
Tyrosine	YFLMLAILNIVFLLGFLVYYYK						√										
Tryptophan	WDAPSRGDDQCQR		√														
Tryptophan	EWLDELK								√								
Tryptophan	EREEDWRQPR			√													
Tryptophan	IKYIETWNPNNQEFQCAGVALSR									√							
Tryptophan	MCCCSWCTCISNWIKMEGSSGSAWQK						√										
Arginine	RRPTEEIEER										√						
Arginine	SLPYSPYSPHSRPR												√				
Arginine	RCNLSPETICHCLFFCQK																√
Arginine	REEEEEEEEER							√									
Serine	SSSSTTFTCCFQR					√											

Note: “√” indicates the detected modification, the underlined and bold letters indicate the corresponding modification sites. AAA = α-aminoadipic acid; AAS = α-aminoadipic semialdehyde; GGS = γ-glutamic semialdehyde; HNE = 4-hydroxy-2-nonenal; MDA = Melondialdehyde.

**Figure A1:**
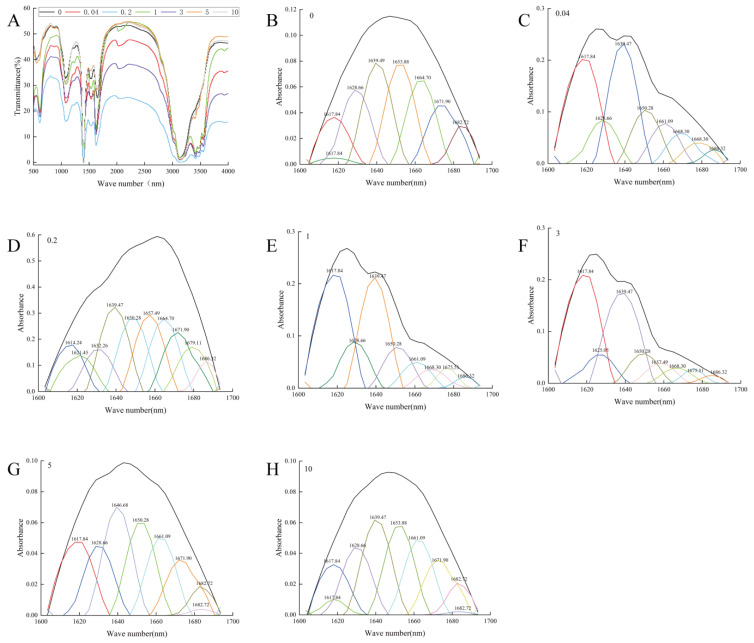
The diagram of fourier transform infrared spectroscopy and secondary structure fitting. (**A**) represents the infrared spectrum. (**B**–**H**) represents the oxidation concentrations of different AAPH, namely 0, 0.04, 0.2, 1, 3, 5, and 10 mmol/L.

## Data Availability

Data are contained within the article.

## Supplementary Materials

The following supporting information can be downloaded at: https://www.mdpi.com/article/10.3390/molecules28176277/s1: Table S1, peptide sequences; Table S2, proteins; Table S3, differentially expressed proteins; Figure S1, proteasome, protein processing in endoplasmic reticulum pathway.

## Appendix A

Infrared spectroscopy is a significant method used to determine the relative content of protein secondary structures. Proteins exhibit numerous characteristic absorption bands in the infrared spectrum. Of particular interest is the amide I band (1600–1700 cm^−1^), which provides valuable secondary structural information about the protein. Figure A1A presents the infrared spectra of the peanut globulin secondary structure under various AAPH oxidation concentrations. Additionally, fitting plots corresponding to these spectra are displayed in Figure A1B–H.
