# Supplementary figures and images for: The Impact of AAPH-Induced Oxidation on the Functional and Structural Properties, and Proteomics of Arachin
Source: Molecules. 2023 Aug 28;28(17):6277. doi: 10.3390/molecules28176277 (PMC10489151; doi:10.3390/molecules28176277)

Figure S1, proteasome, protein processing in endoplasmic reticulum pathway

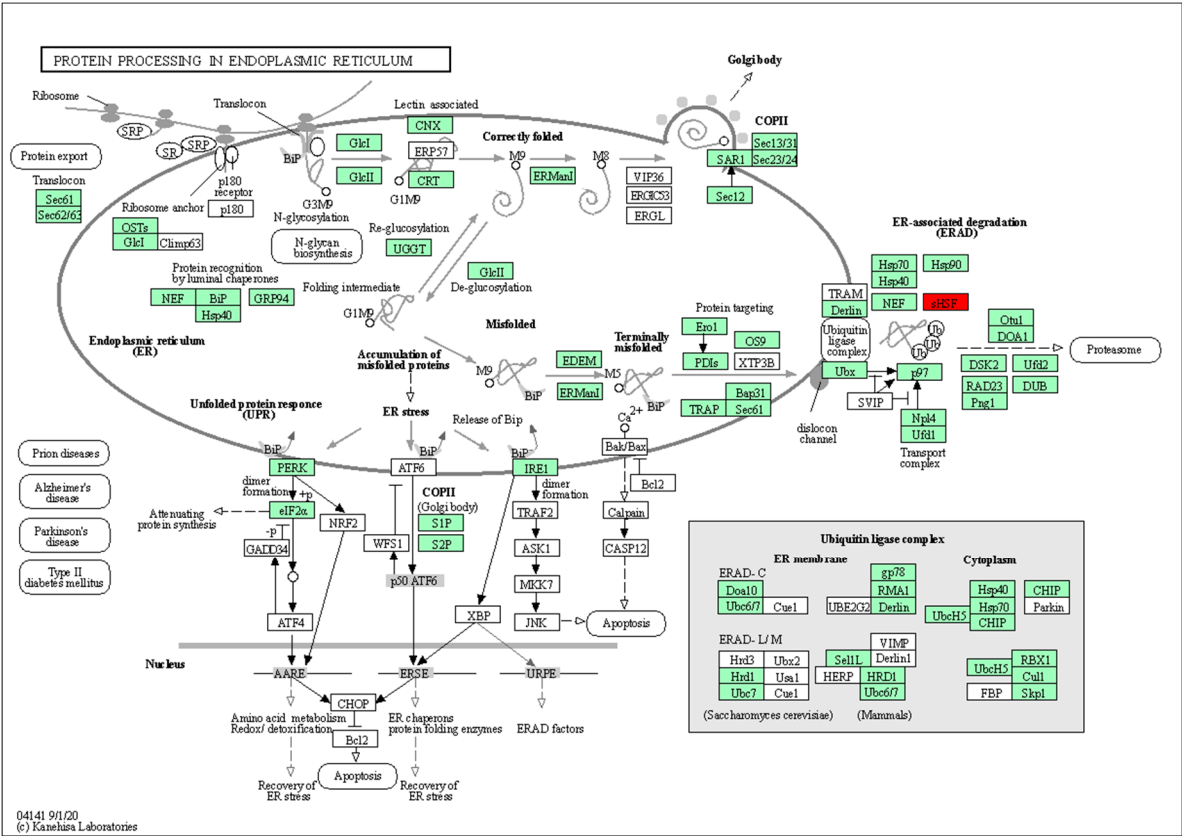

Supplement: Supplementary file 1 [file molecules-28-06277-s001.zip › molecules-2556158-Figure S1.pdf]
